# First implementation of an innovative infra‐red camera system integrated into a mobile CBCT scanner for applicator tracking in brachytherapy—Initial performance characterization

**DOI:** 10.1002/acm2.14364

**Published:** 2024-04-16

**Authors:** Andre Karius, Lisa Marie Leifeld, Vratislav Strnad, Rainer Fietkau, Christoph Bert

**Affiliations:** ^1^ Department of Radiation Oncology Universitätsklinikum Erlangen, Friedrich‐Alexander‐Universität Erlangen‐Nürnberg (FAU) Erlangen Germany; ^2^ Comprehensive Cancer Center Erlangen‐EMN (CCC ER‐EMN) Erlangen Germany

**Keywords:** brachytherapy, combined camera‐CT system, infra‐red applicator tracking, system characterization

## Abstract

**Purpose:**

To enable a real‐time applicator guidance for brachytherapy, we used for the first time infra‐red tracking cameras (OptiTrack, USA) integrated into a mobile cone‐beam computed tomography (CBCT) scanner (medPhoton, Austria). We provide the first description of this prototype and its performance evaluation.

**Methods:**

We performed assessments of camera calibration and camera‐CBCT registration using a geometric calibration phantom. For this purpose, we first evaluated the effects of intrinsic parameters such as camera temperature or gantry rotations on the tracked marker positions. Afterward, calibrations with various settings (sample number, field of view coverage, calibration directions, calibration distances, and lighting conditions) were performed to identify the requirements for achieving maximum tracking accuracy based on an in‐house phantom. The corresponding effects on camera‐CBCT registration were determined as well by comparing tracked marker positions to the positions determined via CBCT. Long‐term stability was assessed by comparing tracking and a ground‐truth on a weekly basis for 6 weeks.

**Results:**

Robust tracking with positional drifts of 0.02 ± 0.01 mm was feasible using the system after a warm‐up period of 90 min. However, gantry rotations affected the tracking and led to inaccuracies of up to 0.70 mm. We identified that 4000 samples and full coverage were required to ensure a robust determination of marker positions and camera‐CBCT registration with geometric deviations of 0.18 ± 0.03 mm and 0.42 ± 0.07 mm, respectively. Long‐term stability showed deviations of more than two standard deviations from the initial calibration after 3 weeks.

**Conclusion:**

We implemented for the first time a standalone combined camera‐CBCT system for tracking in brachytherapy. The system showed high potential for establishing corresponding workflows.

## 1 INTRODUCTION

Since the introduction of three‐dimensional (3D) imaging for brachytherapy in the late 20th century,^1^, ^2^ modalities such as computed tomography (CT) and cone‐beam CT (CBCT) have been established as an essential part of many treatment workflows.^1^, ^2^, ^3^, ^4^, ^5^, ^6^ The cross‐sectional visualization of the patient's anatomy enables not only the delineation of diseases but also a pre‐planning of interventional procedures.^1^, ^2^, ^7^, ^8^, ^9^, ^10^

The implantation of brachytherapy applicators is commonly performed based on imaging. For instance, catheter insertion for breast brachytherapy can be controlled by radiography.^11^ For permanent and temporary prostate brachytherapy, ultrasound imaging is the gold‐standard and enables real‐time needle guidance.^2^, ^6^ Transrectal or transabdominal ultrasound also finds application for brachytherapy of cervical cancer.^12^, ^13^, ^14^, ^15^ However, for regions deep in the pelvis (e.g., at the fundus uteri or parametrium) ultrasound may not provide adequate visualization for real‐time image guidance.^16^, ^17^, ^18^, ^19^, ^20^ For this reason, several workflows focusing on intraoperative magnetic resonance imaging (MRI), CT, or CBCT in lithotomy position for applicator positioning control have been proposed.^17^, ^18^, ^21^ However, these modalities require the patient to be moved from the lithotomy position to the legs down to fit inside the gantry or bore, and the medical staff to leave the surgery hall or intervention room during image acquisition. Another option is infra‐red (IR) guided brachytherapy, as described in the present work, which may allow physicians to make real‐time implant adjustments during the procedure, rather than retrospectively modifying the implant based on the acquired CT or MRI scans. Additionally, it can reduce the imaging dose from repeated CT examinations that may be required (depending on the case) to create a sufficient applicator arrangement.

To avoid multiple scans and provide real‐time guidance during the interventional brachytherapy procedure, our aim is to track rigid applicators and metal needles, that have to be attached to a tracking tool equipped with IR markers, during the implant procedure. For a rigid applicator or needle (i.e., assuming a known rigid relationship between the markers and the applicator/needle tip), tracking the marker tool located at the distal portion of the applicator outside of the patient can be used to predict the applicator location inside the patient in real‐time as an alternative to ultrasound. In this way, the applicator course can be visualized in situ, e.g. on a single, initially acquired CBCT scan. This enables the guidance of implantations even in cases where ultrasound imaging is not sufficient. It must be noted that the proposed method requires a high applicator rigidity and is therefore limited to stable applicators such as tandem or Fletcher as well as metal needles.

To achieve this goal, we used for the first time two IR‐cameras directly integrated into a mobile CBCT scanner to provide a robust basis for tracking and position transfer into the CBCT coordinate system. The present work provides a first technical description of this integrated device as well as its performance evaluation to investigate capabilities in absolute tracking for brachytherapy. We evaluated the effects of intrinsic parameters such as camera temperature and gantry rotations on tracking quality, and identified calibration requirements to achieve maximum tracking accuracy.

## 2 MATERIALS AND METHODS

### 2.1 System description

To facilitate adaptive brachytherapy for cervical cancer using 3D intraoperative imaging, our institution acquires CBCT scans with a mobile CBCT scanner (ImagingRing m, medPhoton, Austria), as previously described.^17^, ^22^, ^23^, ^24^ The device features a gantry with 121 cm clearance and a 43.2 × 43.2 cm^2^ flat‐panel detector. A battery‐mode allows translations and rotations of the mobile scanner on the floor, as well as tilting of its gantry. Following these descriptions, the ImagingRing enables acquisitions in lithotomy position as well as mobile imaging.

**FIGURE 1:**
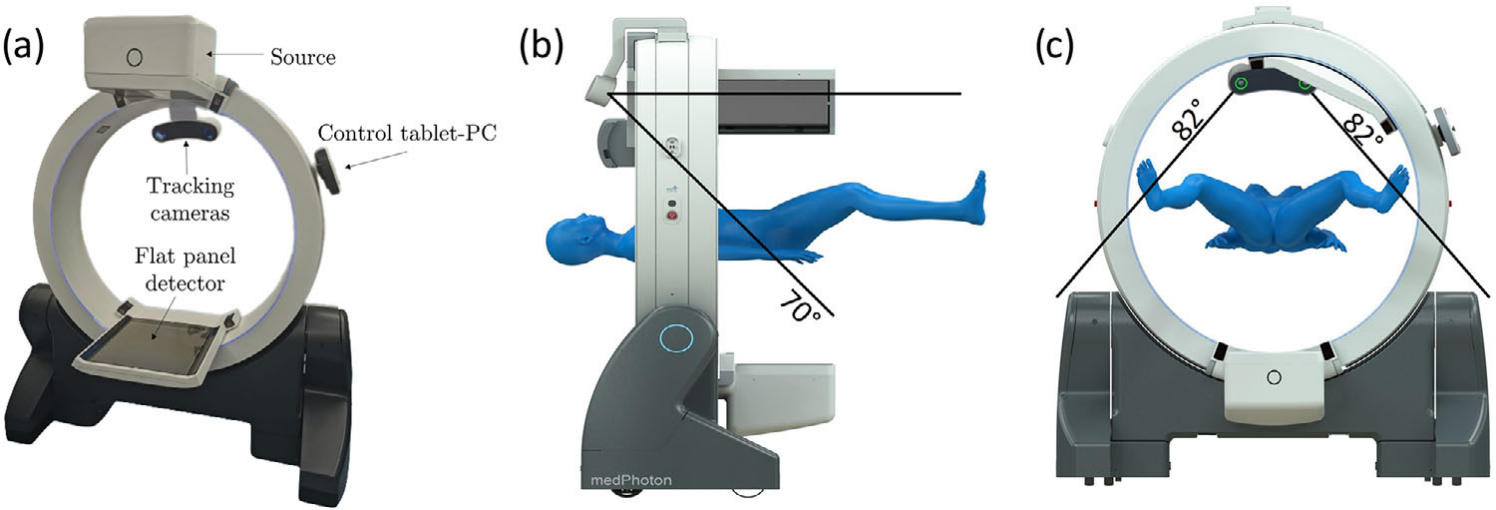
Shown is the setup of the standalone camera‐CBCT system, which integrates two infra‐red tracking cameras into the mobile ImagingRing m (a). The cameras enable tracking in a field of view of 70° and 82° in vertical and horizontal direction, respectively (b,c; graphics provided by medPhoton).

The manufacturer of the ImagingRing provided a gantry‐mounted assembly with two IR‐cameras to create a standalone combined camera‐CBCT system. In this work, we explored the cameras’ (Figure 1a) capability regarding absolute tracking for the purpose of applicator‐guidance in brachytherapy. The tracking system consists of two Prime^x^ 13 W cameras (OptiTrack, USA) with integrated IR emitter and receiver, which define a field of view (FOV) of 82° in the horizontal and 70° in the vertical direction (Figure 1b,c). The cameras feature a resolution of 1.3 MP each with an imaging uncertainty of 0.2 mm (specified by the manufacturer) and a native frame rate of up to 240 fps, and can detect both active and passively reflected IR signals of 850 nm wavelength (e.g., from IR markers). The system is operated through the Motive software (version 3.0.3, OptiTrack, USA), which determines the positions of tracked markers within a 3D camera coordinate system using stereo‐triangulation.^25^

### 2.2 Camera calibration

Each individual IR camera provides a two‐dimensional (2D) position for every tracked marker. To compute the marker position in 3D space from the 2D information, a calibration of the stereo‐system comprising both cameras has to be conducted.^25^ The aim of the calibration is to create a 3D space model that is free from distortions, warping, or misalignment based on the recordings of a wand calibration tool (see below), as described in detail in reference 25.

**FIGURE 2:**
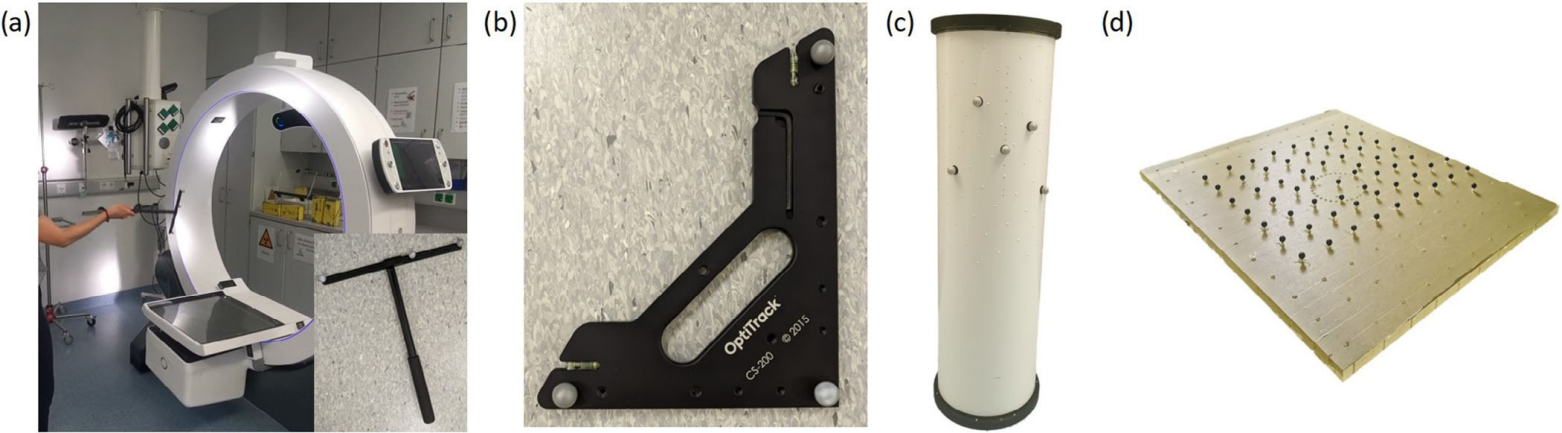
To calibrate the system, the CWM‐250 calibration wand (OptiTrack, USA) is waved in front of the cameras (a). The camera coordinate system origin is then set using the CS‐200 calibration square (OptiTrack, USA) (b). Camera‐CBCT registration is performed using the flexmap‐phantom^26^ with five attached infra‐red markers, which is placed in the gantry center (c). Calibration accuracy was evaluated by means of an in‐house plate phantom comprising 60 infra‐red markers (d).

In this work, calibration is performed using the CWM‐250 calibration wand (OptiTrack, USA; Figure 2a), which consists of three IR‐reflecting markers with known geometric relationships. The 2D positions of the markers captured by each camera are automatically merged to match the known marker relationships of the wand in 3D within the Motive 3.0.3 software. The software also detects varying wand positions over time, each referred to as a “sample” in the following. For the calibration process, several samples have to be recorded by waving the wand in front of the cameras within their combined FOV. This is required to ensure a robust data basis for calculating the 3D coordinate system model. Finally, the origin and ground plane of the camera coordinate system is defined using the CS‐200 calibration square (OptiTrack, USA; Figure 2b), as described in detail in reference 25.

### 2.3 Camera‐CBCT registration

In order to visualize the information associated with marker positions (e.g., the course of rigidly attached applicators) on CBCT scans, registration between the camera and CBCT coordinate systems is required. For this procedure, a geometric calibration phantom (the flexmap‐phantom^22^, ^26^ equipped with five IR markers; Figure 2c) is utilized and has to be placed in the isocenter of the ImagingRing.

Each marker is then tracked by the cameras as well as determined on a corresponding CBCT scan (0.5 × 0.5 × 0.5 mm^3^ voxel size) by using a threshold‐based centroid detection. By applying the Iterative Closest Point (ICP) algorithm,^27^ the marker positions m_1_ tracked by the camera were rigidly registered to the CBCT positions m_2_. This provides a 4 × 4 transformation between the two coordinate systems including rotational (r) and translational (t) components for all spatial dimensions x, y, z:

(1) $$\left(\begin{array}{c} m_{2,x}\\ m_{2,y}\\ \begin{array}{c} m_{2,z}\\ 1 \end{array} \end{array}\right)=\left(\begin{array}{cccc} r_{xx} & r_{xy} & r_{xz} & t_{x}\\ r_{yx} & r_{yy} & r_{yz} & t_{y}\\ r_{zx} & r_{zy} & r_{zz} & t_{z}\\ 0 & 0 & 0 & 1 \end{array}\right)\;\cdot \left(\begin{array}{c} m_{1,x}\\ m_{1,y}\\ \begin{array}{c} m_{1,z}\\ 1 \end{array} \end{array}\right).$$

This transformation provides for all marker positions tracked by the cameras the corresponding positions within the CBCT coordinate system.

### 2.4 System evaluation

The calibration and registration process can be affected by several parameters. In particular, the number of recorded samples and the FOV coverage with these samples are considered critical for achieving a high tracking accuracy. In addition, parameters such as the wand waving direction or the distance between wand and cameras can affect the establishment of a rigid 3D coordinate system. Environmental factors such as camera temperature, gantry rotations, and lighting conditions might impact deviations from the calibration as well. Since our work was the first to systematically evaluate the combined camera‐CBCT system for absolute tracking, no data regarding these effects or a description of the minimum calibration and quality requirements were previously available.

For this reason, the effects of variations in the aforementioned parameters on the resulting calibration and tracking quality were investigated. Based on initial assumptions, a baseline calibration (shown in Table 1) was established. Each individual calibration parameter was then varied as listed in Table 1, while keeping the other parameters fixed, to investigate the effects of these specific variations. In each case, the tracking and calibration quality were evaluated as follows.

**TABLE 1:** Parameters of the baseline calibration and all parameter variations conducted in this work.

Parameter	Baseline	Variation
Samples	1000	50, 200, 1000, 2000, 4000, 6000, 8000, 10000
Wand waving direction	All spatial directions randomly	Horizontally only, vertically only, all spatial directions randomly
Wand coverage	Full field of view (FOV) coverage	Left FOV half only, right FOV half only, top FOV half only, bottom FOV half only, full FOV coverage
Wand‐camera distance	Varying distance between 0.2 m and 2 m	0.2 m distinct, 2 m distinct, varying distance between 0.2 m and 2 m
Light conditions	Room light only	Room light only, Room light off + surgical lamp, room light + surgical lamp, room light + switching surgical lamp, room light off + switching surgical lamp

#### 2.4.1 Camera characteristics

In the first step, the effect of camera temperature and gantry position on marker tracking was evaluated. To achieve this, the baseline calibration was applied and the flexmap‐phantom was placed in the isocenter of the ImagingRing.

For the assessment of temperature effects, the phantom markers were tracked every 30 s for a duration of 2 h, starting immediately after switching on the device. For each marker and time point t, the Euclidean distances to the positions tracked at the previous time point were calculated. Tracking was considered stable if no position changes (<0.2 mm) occurred over time.

To evaluate the effect of gantry rotations on tracking, the flexmap‐phantom remained centered within the ImagingRing, and a 360°CBCT scan with an acquisition time of 96 s was performed. The long scan time was chosen to achieve a high angular sampling and to obtain a large amount of data during the 360° rotation. For each scan, the marker positions were tracked simultaneously to the acquisition of the planar CBCT projections (the flat‐panel was operated with 12 Hz frame rate), i.e. every 0.08 s. The Euclidean distances to the initially tracked positions were then calculated as a function of time and gantry angle. This measurement was performed for 10 independent 360° scans in succession.

#### 2.4.2 Calibration quality

To evaluate the calibration procedure, the individual calibration parameters listed in Table 1 were varied while all other parameters were kept at the baseline. Each of the resulting individual calibrations was performed three times.

To assess tracking accuracy for each calibration, 60 IR‐markers were attached with a spacing of 5 cm (with three additional markers in one corner and one omitted marker in the center to create an asymmetry) onto an acrylic plate with dimensions of 40 × 40 cm^2^ (Figure 2d). The plate was covered with tape to avoid reflections. A CT scan with 0.3 × 0.3 × 1 mm^3^ voxel size was acquired using a SOMATOM go.Open Pro (Siemens Healthineers, Germany) scanner, and the marker centroids were detected threshold‐based to obtain the ground‐truth marker arrangement. The plate was then positioned both horizontally (i.e., flat‐lying) and vertically on the table at a distance of 0.75–1 m from the cameras and at the height of the gantry center, to cover almost the entire camera FOV. For each calibration and both plate settings, IR‐tracking was performed three times. In each case, the Euclidean distances $e_{i,j}$ of each tracked marker i to all other markers j were determined. The respective differences $\Delta_{i,j}$ to the ground‐truth were calculated for all combinations i,j as measure of the location‐dependent geometric tracking fidelity.

(2) $$\Delta_{i,j}\;=\;e_{measurement}\left(i,j\right)-\;e_{ground-truth}\left(i,j\right)\;\forall \;i,j\;\left(i\neq j\right).$$

#### 2.4.3 Registration quality

For evaluating the impact of calibration quality on the camera‐CBCT registration, a coordinate system registration according to Section 2.3. was performed after each calibration. The root‐mean‐square‐deviation (RMSD) of the marker positions tracked and transferred into the CBCT system to those measured directly via CBCT was calculated. This served as a measure of the registration accuracy achievable with our integrated system.

#### 2.4.4 Stability over time

The stability of calibrations is important to ensure an accurate tracking over time. In the following, the CBCT coordinate system is considered to be fixed due to the previously observed^23^ good long‐term stability of the ImagingRing. Therefore, tracking errors may arise mainly from variations in the camera coordinate system. In order to detect such variations, the plate measurements are performed in Section 2.4.2. were repeated weekly over a period of 6 weeks under the application of this specific calibration that turned out to provide the highest quality (considering the results of Sections 2.4.1–2.4.3.). This calibration was performed only once at the beginning of the 6‐week period. Six weeks was chosen since the camera quality assurance (QA) was intended to be integrated into the ImagingRing QA conducted monthly. Therefore, the variations occurring in this time frame were of high interest for identifying the required re‐calibration frequency. Note that the ground‐truth marker arrangement was re‐calculated based on a CT prior to each measurement to account for potential changes caused by, for example, screw bending.

## 3 RESULTS

### 3.1 Camera characteristics

**FIGURE 3:**
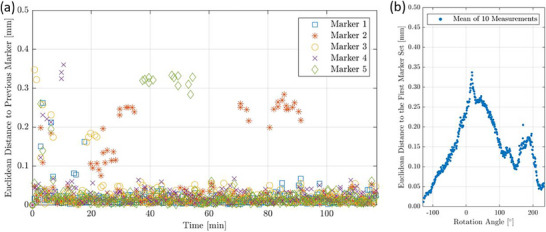
Shown are the results of the marker tracking measurements conducted for assessing the temperature dependence of the tracking process. For each of the five tracked markers and each time point, the Euclidean distance to the tracked positions at the previous time point is provided, revealing the impact of camera temperature (a). Furthermore, the effect of gantry rotations on the tracked centroid of the five markers (averaged over all 10 measurements) relative to its position tracked prior to the start of the rotation is shown. The individual measurements are not shown here for clarity. The gantry rotations affected the tracking process and the determined marker positions (b).

The measurements on camera characteristics revealed that the tracking procedure was affected by the varying camera temperature throughout the system warm‐up. Deviations of the tracked marker positions were enhanced in an early camera warm‐up phase of up to 35 min with position variations of up to 2.0 mm compared to the initial marker positions and position uncertainties of up to 0.36 mm (Figure 3a). Starting from 90 min after switching on the cameras, an equilibrium state was reached with variations of about 1.9 mm from the initial marker positions, and no positional outliers occurred anymore. In this state, position changes showed a standard deviation of 0.02 ± 0.01 mm. Thus, with the current camera implementation, conducting a warm‐up of about 90 min was important to ensure robust tracking.

Furthermore, for warmed‐up cameras, we found an impact of varying gantry rotation angles on tracking. Position deviations of up to 0.70 mm and with a mean of 0.15 ± 0.09 mm (averaged over all angles and measurements) were obtained (Figure 3b). The found variations thus exceeded the camera uncertainties and were related to the gantry rotation process. To achieve accurate results, it appeared important to perform tracking with the same gantry position that was set for calibration.

### 3.2 Calibration quality

**FIGURE 4:**
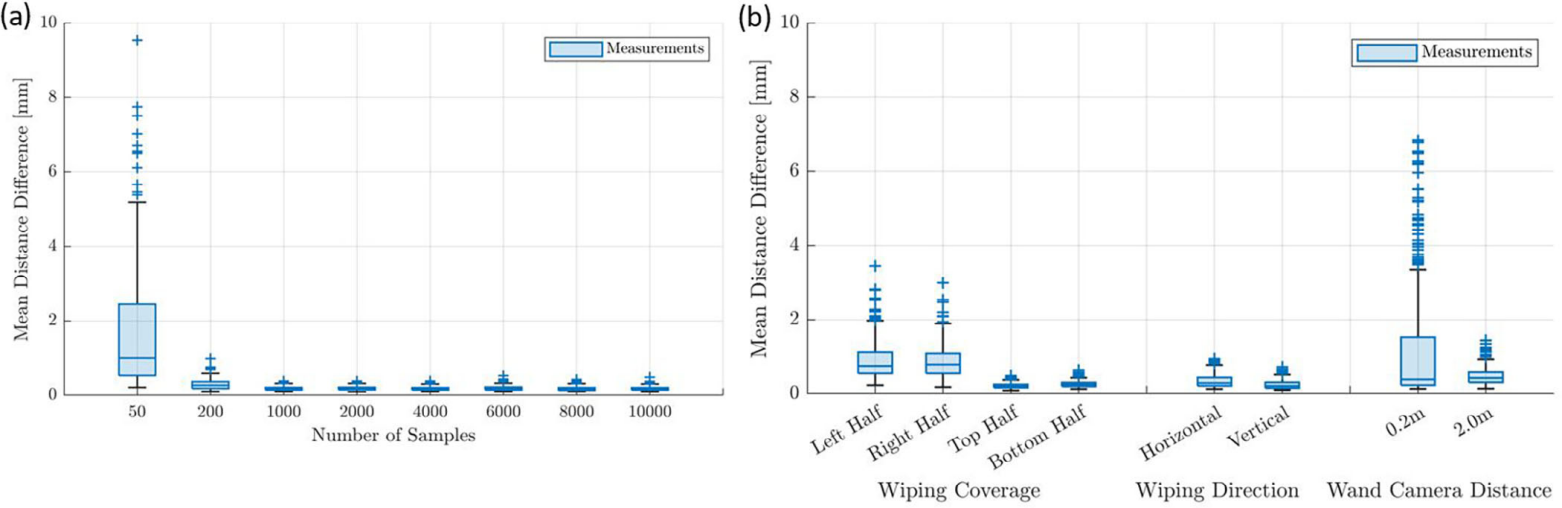
Shown is the effect of varying the number of samples on tracking accuracy, i.e. the marker distance deviations from the ground‐truth measured for all markers, that were obtained in the plate measurements (a). Furthermore, the results obtained for varying wand waving directions, field of view coverage, and wand‐camera distances are provided (b).

In analyzing the impact of the individual calibration parameters, we found that the number of samples recorded affected the tracking accuracy. As shown in Figure 4a (which includes both considered plate orientations), the calibration performed with 50 samples revealed geometric deviations from the ground‐truth marker arrangement of up to 9.54 mm. The locations of large discrepancies were not fixed and depended on the individual calibration. Geometric deviations decreased as the number of samples increased until a recorded number of 1000 was reached. For this setting, we found a maximum and mean deviation of 0.52 mm and 0.18 ± 0.03 mm, respectively. Only minimal differences were observed between the calibrations performed with ≥1000 samples, and no relevant discrepancies between the two plate orientations were obtained in this range. A sample number of at least 1000 was therefore required to achieve a high calibration quality.

The sample coverage of the FOV, wand‐waving direction, and wand‐camera distance affected the tracking accuracy as well (Figure 4b). For instance, calibrations with the wand being waved only in the left or right part of the camera FOV resulted in reduced geometric accuracy with maximum deviations of up to 3.45 mm and 2.99 mm, respectively. Covering only the upper or lower parts of the FOV yielded improved results with maximum deviations of 0.48 mm and 0.65 mm and mean values of 0.22 mm and 0.27 mm, respectively. This was worse than the results obtained for the baseline calibration performed with full FOV coverage (see above). Reducing the wand‐waving to the horizontal or vertical direction also resulted in increased inaccuracies of up to 0.95 mm and 0.73 mm, respectively. As a consequence, ensuring full FOV coverage with ≥1000 samples and considering a variety of arbitrary wand‐waving directions was required to achieve optimal tracking accuracy.

In addition, samples should be recorded at varying distances from the camera throughout the entire tracking region of interest. This is since calibrations restricted to specific distances led to a reduced accuracy, with maximum geometric deviations of up to 6.84 mm (at 0.2 m) and 1.44 mm (at 2 m), respectively. The location of larger deviations was again not fixed but depended on the calibration. Finally, varying light conditions had no effect on calibration quality.

### 3.3 Registration quality

**FIGURE 5:**
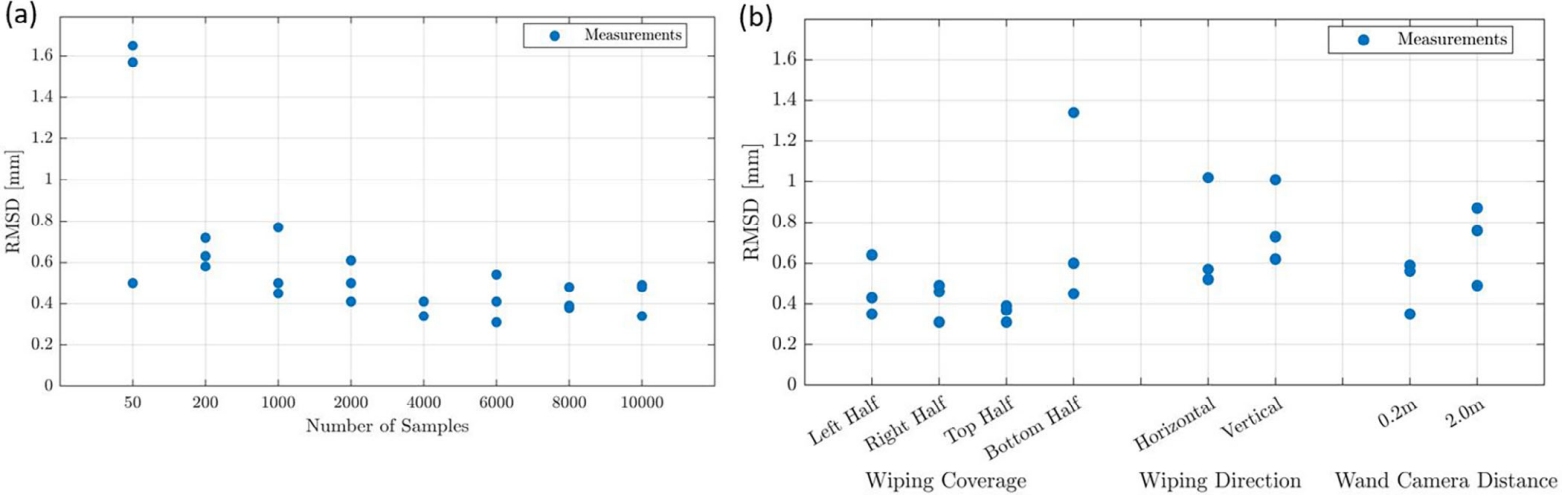
Shown is the effect of varying sample numbers on the resulting root‐mean‐square‐deviations (RMSDs) obtained for the individual camera‐CBCT registration procedures (a). In addition, the results obtained for varying wand waving directions, field of view coverage, and wand‐camera distances are provided (b).

Investigating the impact of the varying calibration parameters on coordinate system registrations, we observed a dependence of the results on the number of samples recorded (Figure 5a). For calibrations performed with ≤2000 samples, we achieved maximum RMSDs of 0.61–1.65 mm. However, for calibrations with ≥4000 samples, registration quality was improved and no relevant differences were found with increasing sample number. For the latter range, we calculated a mean RMSD of 0.42 ± 0.07 mm with a maximum of 0.54 mm. Thus, the recording of at least 4000 samples was considered critical to achieve both maximum tracking and registration accuracy. In this sample range, the RMSDs were in the order of magnitude of the geometric deviations found in the calibration quality assessment (Section 3.2.). Note that the recording of 4000 samples took only about 8 min, meaning that this requirement could be fulfilled within a reasonable time frame.

Calibrations performed with varying wand waving directions, FOV coverages, and distinct distances from the cameras affected registration quality as well (Figure 5b). The RMSD achieved by the baseline calibration (with an increased sample number of 4000 instead of 1000) could only be obtained by the calibration where only the upper (0.36 ± 0.05 mm) or the right (0.42 ± 0.10 mm) part of the FOV was covered, respectively. All other calibrations showed worse results with a maximum RMSD of at least ≥0.6 mm and partly even ≥1 mm. Different lighting conditions did not affect the registration quality.

### 3.4 Stability over time

**FIGURE 6:**
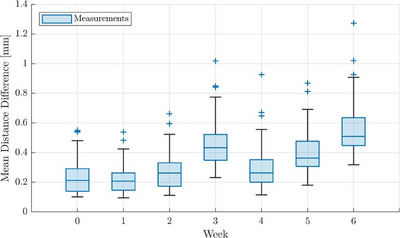
Shown are the results obtained regarding the system's long‐term stability, i.e. in assessing the variations of marker distance deviations compared to the ground‐truth, that were measured for all markers over a duration of 6 weeks.

To assess long‐term stability, the calibration associated with the highest tracking accuracy (i.e., the baseline calibration with 4000 samples instead of 1000) was considered. Based on this, we observed geometric marker deviations that exceeded the day 0 calibration results (reported above) by more than two standard deviations from the third week onward, with variations of about 0.45 ± 0.14 mm (Figure 6). A general trend of decreasing tracking accuracy over time was observed. For instance, we found maximum deviations of 0.54 mm, 0.66 mm, and 1.02 mm for the first, second, and third week after the calibration, respectively (Figure 6). Therefore, regular QA and re‐calibration of the cameras seemed inevitable to ensure high tracking quality over time.

## 4 DISCUSSION

An innovative IR‐camera system has been directly integrated into a mobile CBCT scanner for image‐guided adaptive brachytherapy for the first time. We conducted a performance characterization of the combined system with respect to calibration and registration quality as a basis for the intended tracking of brachytherapy applicators. With the best calibration settings of full FOV wand coverage in all directions and 4000 samples, a geometric tracking and registration accuracy of x = 0.18 ± 0.03 mm and y = 0.42 ± 0.07 mm was obtained, respectively. The average positional fidelity of the markers within the CBCT coordinate system, which were tracked by the cameras and transferred accordingly, can therefore be specified to $\sqrt{x^{2}+y^{2}}=$ 0.46 ± 0.28 mm at maximum. Taking into account potential CBCT partial volume effects and camera measurement uncertainties, the achieved result is considered sufficient for tracking brachytherapy applicators and corresponds to the upper accuracy limit with which applicators attached to markers can be tracked by the system.

Tracking of applicators gains rising importance in brachytherapy,^28^, ^29^, ^30^ due to image‐guidance in general becoming an essential component of many brachytherapy workflows in recent years.^31^, ^32^, ^33^, ^34^ In this respect, one aim is to adapt the implantation procedure to the patient with high accuracy. However, a real‐time image‐guidance is currently only feasible with fluoroscopy and ultrasound, both concomitant with insufficient visualization of the anatomy of interest in some cases. For instance, in brachytherapy for cervical cancer, especially regions deep in the pelvis may not be accessible by standard ultrasound techniques.^16^, ^17^, ^18^, ^19^, ^20^ In this regard, Knoth et al.^16^ were only able to detect 87% and 51% of the implanted straight and oblique needles, respectively, using transrectal ultrasound. Therefore, intraoperative MRI or CBCT associated with increased effort, as described in the introduction, is often the method of choice.^17^, ^18^, ^21^ The implantation can then be performed based on the acquired images, but not guided with real‐time feedback to avoid potential injury to organs at risk. To address this issue, our aim is to track applicators using IR‐cameras and to predict their course in‐situ in one single initially acquired CBCT scan.

Several approaches for tracking in interventional and surgical medicine exist in general.^28^, ^29^, ^30^, ^35^ One possibility is electromagnetic tracking (EMT), in which the magnetic field of field generators is perturbed by corresponding sensors, allowing for conclusions about the sensor positions in 3D space.^30^, ^36^, ^37^, ^38^, ^39^ However, to project these positions into a CBCT scan, an additional device tracking both the field generator and the CBCT scanner would be required for a respective coordinate system registration.^37^ This is associated with higher uncertainties than a direct camera‐CBCT registration as conducted in the present work. An alternative is optical or IR tracking applied many times in radiotherapy, e.g. for patient positioning at the linear accelerator^40^, ^41^, ^42^ or four‐dimensional CT.^43^, ^44^ For interventions and surgery, such tracking was also already used for (pre‐)clinical investigations.^45^, ^46^, ^47^ However, corresponding approaches are mainly based on external tracking cameras that would again require a tracking of both CBCT scanner and actual marker tool to establish a position transfer, concomitant with increased inaccuracies of up to >2 mm reported.^35^, ^48^ It should be noted that a direct comparison between different tracking approaches is complex and has to be performed for the specific medical task under investigation. A comprehensive review on this topic (including both electromagnetic and optical tracking) pointing out the various (dis)advantages has previously been published by Sorriento et al.^49^ Nevertheless, a standalone integrated camera‐CBCT system for brachytherapy, which only requires the tracking of a marker tool to project positional information into the CBCT coordinate system (due to the rigid camera‐CBCT relation), has been implemented and characterized for the first time in the context of this work. To our knowledge, standardized real‐time applicator tracking during interventional procedures has not yet been implemented for brachytherapy.

The results of this study are relevant not only for achieving optimal tracking quality with our combined system but also for infrared (IR) tracking systems in general. For instance, we demonstrated the importance of accounting for camera temperature and its impact on geometric accuracy. In clinical operation, it will be important to warm up the cameras of our system for up to 90 min prior to being used. This means that the cameras must be switched on at least 90 min prior to a corresponding surgical procedure, with no further manual user interaction being required. Furthermore, the impact of gantry positions on marker position determination was identified. In the current state, it is necessary to perform tracking with a fixed gantry position, as established during calibration, to ensure optimal tracking precision. During gantry rotations, wobbling of the CBCT scanner^22^, ^23^ or mechanical hysteresis effects^22^ might result in varying stress to the system, which is transferred to the camera mounting due to its rigid assembly. A further calibration procedure similar to the ImagingRing's flexmap calibration,^26^ which measures tracked marker positions as a function of the gantry angle and accounts for occurring spatial deviations during live‐tracking, could be beneficial to improve system performance.

We found that a minimum number of 4000 samples was required to achieve a high calibration and registration quality, although 1000 samples were sufficient to establish a reasonable camera coordinate system. One explanation could be that the flexmap‐phantom must be positioned in the gantry center and thus close to the cameras, whereas the plate measurements were conducted at a larger distance referring to the main clinical operation area. In this respect, 1000 samples are considered beneficial for achieving a high geometric accuracy in this central region but also associated with slight deteriorations at the longitudinal FOV borders. A higher number of 4000 samples better accounts for this issue and ensured a good registration quality with an RMSD of 0.42 ± 0.07 mm. Only when both tracking and camera‐CBCT registration are optimized, an accurate transfer of marker positions into the CBCT system becomes feasible. Observations of a reasonable registration quality in some of the scenarios that revealed a reduced calibration quality can be explained by these calibrations leading at least to a preservation of the geometric relations of the five centered flexmap‐phantom markers.

Furthermore, we showed that a high sample coverage of the entire camera FOV in all spatial directions is essential to achieve a good tracking performance. However, the direction horizontal to the ground seemed to be more important to create a suitable 3D space model. This is probably due to the cameras being arranged horizontally to the ground and thus the primary necessity to cover this direction to enable a sufficient stereo‐triangulation.^50^, ^51^ Varying lighting conditions did not affect our measurements, but this is considered an environmental issue and should be investigated separately for each application setting.

Regarding long‐term stability, we observed increased position deviations of the tracked markers starting about 3 weeks after the calibration. Our results highlight the need for a regular camera QA routine and re‐calibration. Semi‐automatic QA procedures ensuring improved stability are aimed at. Fixed thresholds triggering re‐calibrations cannot be provided yet but have to be determined based on the results of pre‐clinical applicator tracking studies. In our institution, we currently plan to determine calibration quality prior to each measurement day and to re‐calibrate the system if the mean geometric marker deviations exceed the results obtained immediately after the original calibration by more than two standard deviations. In the present work, this set threshold of two standard deviations was exceeded after 3 weeks (according to the results described in Section 3.4.), indicating that regular re‐calibrations may be required at least once a month. One reason for the observed low long‐term stability could be a performance degradation of the cameras. However, it is more likely that the mobility of the ImagingRing, with many daily movements for CBCT imaging in clinical routine,^17^ could lead to geometric changes of the camera relations, which has to be examined in further studies.

Despite the high tracking accuracy already achieved, it should be noted that we have performed an initial performance characterization of the prototype system, and further improvements are to be expected. The determined maximum tracking accuracy represents the upper limit achievable with the system as currently implemented, but the actual geometric fidelity of the marker positions transferred into the CBCT coordinate system may be lower. This is since we investigated registration quality by means of the flexmap‐phantom centered in front of the cameras, but even small rotational errors in the registration process may lead to larger discrepancies between transferred and actual marker positions at larger distances from the coordinate system origin. The applicator tracking accuracy achievable in clinical routine therefore remains to be determined in initial phantom studies and is expected to be lower than the maximum accuracy provided. Nevertheless, to our knowledge, the combined camera‐CBCT system sets new standards regarding tracking for brachytherapy. Most importantly, our work reports explicit minimum calibration requirements for setting up the system and achieving a good tracking performance.

It should be reiterated that the focus of the present work was on an initial pre‐clinical performance evaluation of the combined device to establish an ideal use workflow. Investigating the feasibility of the device to track applicators and needles in (pre‐)clinical scenarios and practice has to form the subject of future investigations, but was beyond the scope of this article. In this context, assessing the accuracy of applicator or needle tip predictions will be most important, considering potential bending in situ during the implantation. In the case of bending, the assumption of a rigid relationship between the tracked marker tool and the applicator/needle tip is no longer valid, which would result in deviations between predicted and actual tip positions. The corresponding clinical relevance and impact on positioning accuracy will need to be determined in further studies. However, since the assumed rigid relationship is a prerequisite for the proposed IR‐tracking, our approach is limited to stable applicators such as tandem or Fletcher as well as metal needles. Since plastic needles are known from clinical experience to be very flexible and to bend more during implantations, we do not plan to consider them for establishing a tracking workflow, which is a limitation of our approach. In any case, the potential effects of bending could be reduced by determining the tip location on ultrasound after the applicator has been inserted to a certain depth, and using IR tracking only for the last few centimeters to be implanted (in regions where ultrasound alone is not sufficient). The clinical workflow would therefore be to use ultrasound imaging as standard imaging modality whenever possible, and to switch to the proposed IR tracking in all cases where sufficient implantation cannot be ensured based on ultrasound. Further logistical requirements, such as the sterility of the marker tool attached to the applicators, have to be defined and fulfilled prior to a final clinical implementation as well.

## 5 CONCLUSION

In the present work, we implemented for the first time a standalone system for CBCT imaging and applicator tracking for brachytherapy. We obtained an upper accuracy limit considering both marker tracking and coordinate system registration of 0.46 ± 0.28 mm, and reported minimum calibration requirements (e.g., recording 4000 samples with full FOV coverage) to achieve maximum performance. The system showed high potential for establishing an applicator tracking, although long‐term stability needs to be improved.

## AUTHOR CONTRIBUTIONS

All authors contributed significantly to the performed work and approved the final version of the manuscript to be published.

## CONFLICT OF INTEREST STATEMENT

The University Hospital Erlangen has research agreements with medPhoton and Elekta regarding ImagingRing m applications in brachytherapy.

## Data Availability

Data are provided by the corresponding author upon reasonable request.
